# A Case Resembling Fungus Hæmatodes Successfully Treated

**Published:** 1840-12

**Authors:** Samuel Henderson


Art. IV.—*A Case resembling Fungus Hæmatodes successfully treated.* Reported to the Medical Society of Tennessee. By Samuel Henderson, M. D.

The following case of Fungus Hæmatodes occurred in the subject of a lady, Mrs. L., ninety-two or three years of age, of full habit, and tolerable health.

Oct. 14th, 1838. I was called to see the patient, who about one year previously discovered a projection from the centre of the forehead, covered with a hard horny scab, which, when removed, left a small fleshy tumor, of a soft, spongy, elastic feel, moveable not discolored, and but slightly painful. Gradually enlarging in size, the old lady become very uneasy. At the time I saw her the tumor had grown to the size of a dollar in diameter, and projecting irregularly half an inch above the surrounding integuments with small openings or fissures over its exterior portion, having the appearance of rough contused, or lacerated edges, of a dark red color yielding to pressure, but soon resuming its former state, and discharging a thin, bloody or ichorous humor, extremely offensive. It had occasionally stinging or darting pains as she described them. Upon examination, we advised extirpation of the tumor, to which she strongly objected. We then applied mild dressings, and pressure by means of a bandage. Afterwards we used escharotics and caustics, which retarded its growth for a time; but when they were omitted, it spouted out like a mushroom. From the extreme uneasiness of the patient, and her great aversion to extirpation, I determined on trying to destroy the substance by some arsenical preparation, with which view its surface was slightly and frequently touched with Fowler's solution. In a few days, the part turned dark, became gangrenous, and sloughed down to the periostium; and by means of mild dressings, and adhesive plasters, the ulcer healed kindly, leaving no trace of disease, except a small ulcer on the nose of the same nature, and two or three small prominences on the face having the appearance of the former.

Will the writer favor the profession with the result of this case? Did the new tumors grow to any thing serious?—*Eds.*

